# Modeling Contact Angles with Chemically Specific Dissipative Particle Dynamics

**DOI:** 10.1021/acs.langmuir.4c04023

**Published:** 2025-02-05

**Authors:** Guadalupe Jiménez-Serratos, Patrick B. Warren, Scott Singleton, David J. Bray, Richard L. Anderson

**Affiliations:** † The Hartree Centre, STFC Daresbury Laboratory, Warrington WA4 4AD, U.K.; ‡ Unilever R&D Colworth Laboratory, Sharnbrook, Bedford MK44 1LQ, U.K.

## Abstract

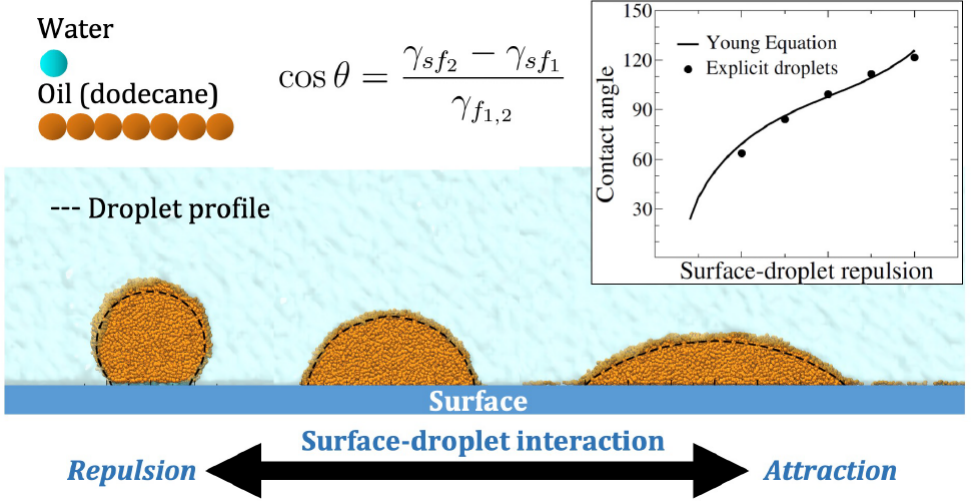

We explore how walls can be introduced into chemically specific dissipative particle dynamics models such that the surface energies can be chosen to obtain a desired contact angle on a given substrate, for example, for an oil/water interface. We certify the methodology for determining the surface energy, which can be positive or negative, such that the Young equation is automatically satisfied. We validate the approach against direct numerical simulation of cylindrical droplets of dodecane in water on the surface and test it against an experimental model of a water droplet in dodecane on a surface-adsorbed monolayer on silica.

## Introduction

Recent developments in dissipative particle dynamics (DPD) allow for the treatment of complex fluids and solutions of surfactants and polymers with chemical specificity, using a coarse-graining or fragmentation strategy in which the DPD “beads” represent chemical groups comprising typically one to three “heavy” atoms (carbon, oxygen, nitrogen, etc.), with the exception of water, which is treated supramolecularly (for details, see below). Although the “force fields” (i.e., the nonbonded pairwise soft repulsions) are parametrized only against liquid phase density data and log *P* values,^1^ these models have proven remarkably capable at capturing for instance the behavior of surfactants, reproducing *inter alia* micelle formation and the critical micelle concentrations for nonionic and ionic surfactants,^2,3^ the properties of surfactant mixtures,^4,5^ and the phase behavior of surfactants,^6^ in a variety of industrially relevant surfactant systems. With additional fine-tuning of the bonded interactions, the models are even capable of capturing the freezing transition (“waxing”) of long-chain alkanes.^7,8^ These developments build capabilities in the area of computer-aided formulation and contribute an essential element to support the industry-wide move away from petrochemicals toward sustainably sourced, ecologically and climate-friendly raw materials confronting what is a huge challenge for the multibillion dollar surfactants business.

Most of the literature in this field is largely focused on the properties of bulk (liquid) materials, with less emphasis based on building substrate models, despite the latter’s perhaps equal importance in numerous practical applications such as in laundry detergency, home and personal care products, emulsion and foam stability, and industrial separation processes. Lin et al.^9^ simulated water droplets onto an explicit surface using many-body DPD (MBDPD) simulations, scanning solid–fluid interactions from hydrophilic to superhydrophobic properties. Patterned surfaces, with applications ranging from printing and spraying processes to nanofluidics have been a common representation in multiple works^10−15^ studying aspects like surfactant adsorption, sliding, and advancing and receding angles, as well as the agreement with the Cassie or Cassie–Baxter equations. Recent works^16,17^ have adopted MBDPD approaches to investigate contact angles of nanobubbles, relevant in wastewater treatment, drug delivery, and nano- or microfluidic research. However, the basic question of how to parametrize substrates in chemically specific DPD models has received scant attention to date. The application of molecular simulations in estimating contact angles has recently been reviewed by Jiang and Patel.^18^

Studies that look to build chemically specific surface models (in DPD) do not yet appear in the literature to the best of our knowledge. Here, we start to lay some foundations in this area by providing the basic building blocks for achieving some degree of specificity.

**Figure 1:**
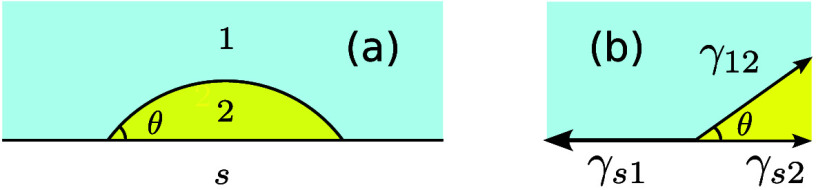
Example of a contact angle (a) and the geometry of the Young equation (b).

The focus of the present study is on the contact angle θ that the interface between two phases (“1” and “2”) forms at the three-phase contact line on a solid substrate (“s”) which is locally flat (Figure 1). Given the interfacial tension between the two phases of interest, γ_12_, and the surface energies of the two phases against the substrate, γ_s1_ and γ_s2_, the contact angle satisfies the Young equation,^19−21^
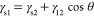
1There are various ways to derive this result (for example, see Supporting Information in section S1), but at its simplest one can view it as a static force balance as indicated in Figure 1b.

For the contact angle to be finite (i.e., 0 < θ < 180°), one must have |γ_s1_ – γ_s2_| < γ_12_. An alternative way to view this is to consider the de Gennes spreading parameter,^20,22^
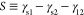
2With this definition, the Young equation can be written
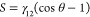
3If we consider phase “2” to be a droplet, as shown in Figure 1a, this makes it clear that there are three domains of behavior,
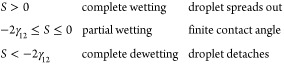
The spreading parameter *S* is, therefore, a measure of the wettability of the substrate by the indicated phases.

Under conditions of partial wetting, the contact angle θ is directly accessible to experiment. As such, it is directly relatable to many of the above-mentioned practical applications: for example, laundry detergents are designed to promote a high contact angle or even complete detachment for an oil droplet on a surface, facilitating disproportionation and roll-up;^23^ antifoams depend on the partial wettability of hydrophobic particles to cause film breakage;^24^ in a Pickering emulsion, colloids or nanoparticles with a judiciously chosen contact angle sit on the interface and stabilize oil droplets;^25^ and in mineral flotation, differential wettability is used to separate the valuable ore from the unwanted “gangue” components.^26^

For coarse-grained simulation methodologies such as our chemically specific DPD models, there are two approaches to modeling walls. The first is simply to extend the paradigm to represent a wall as an array of DPD beads which are either “frozen” or tethered to fixed sites.^11,27^ In this, one can take advantage of the bead–bead interactions already derived within a standard parametrization strategy, but on the other hand the determination of the surface energies may be delicate. Additionally, there is the need to choose the density and arrangement of the beads and parametrize the tethering force if the beads are not frozen. Such an approach could, however, be suitable for materials which are chemically patterned or have nanoscale roughness. An alternative approach, which is the focus here, is to model the surface as a smooth, featureless substrate. This approach is suitable for atomistically or molecularly smooth materials, such as glass (silica), many plastics (for example, polythene, polypropylene, polyester), minerals (calcite, hydroxyapatite, etc.), organic crystals such as sugars (sucrose), and perhaps even ice. Within this approach, which pertains to many situations of direct practical relevance, the parametrization problem becomes simply one of designating the wall forces for each bead type in the bulk. Moreover, determination of the surface energies is very straightforward, as we shall see.

Whichever wall model is adopted, our central thesis is that it should recover the macroscopic contact angle(s) for droplets of given liquids on the substrates of interest, and at the same time, it should minimally perturb the liquid structure on length scales beyond the immediate vicinity of the wall. The reasoning behind the first requirement is that in the absence of directly measured surface energies contact angles stand in for the wettability of the substrate. For example, in saying that a surface is hydrophilic or hydrophobic one is really making a statement about the contact angle of a droplet of water on that surface (while the present work does not cover vapor–liquid interfaces, the methods we develop should be equally applicable). For the second requirement, we note that if a coarse-grained DPD water bead represents several actual water molecules, structure on the bead length scale induced by the wall (e.g., oscillations in the bead density) should be minimized. This latter requirement involves an inevitable trade off in being able to set surface energies arbitrarily, but as a design criterion it can be used to narrow the choice of wall interaction potentials as described below, and take advantage of a built-in degree of freedom since in determining contact angles it is only the *difference* in surface energies, γ_s1_ – γ_s2_, that is relevant for the Young equation and the de Gennes spreading parameter.^20^

In this article, we outline the chemically specific DPD model that we use as a basis and describe the wall model that we use. We then introduce the methodology for calculating the surface energies from our DPD simulations, such that the Young equation is automatically satisfied. Next, we validate this against direct numerical simulations of oil droplets on surfaces using dodecane in water at 25 °C as our model. Finally, we consider the complementary case of a water droplet in oil and show that our methodology can reproduce the case of a water droplet in dodecane on a surface-adsorbed monolayer on silica.

## Materials and Methods

### Chemically Specific DPD Model

We adopt the DPD parameters from Anderson et al.^1^ In this model, DPD bead represents a chemical group comprising a small number of “heavy atoms” (e.g., carbon, oxygen), except for water, which is treated supermolecularly, with the DPD water bead representing two water molecules (2H_2_O). Dodecane (C_12_H_26_) is represented as a 7-mer, CH_3_–(CH_2_CH_2_)_5_–CH_3_, consisting of CH_2_CH_2_ and terminal CH_3_ beads. Note that under atmospheric pressure dodecane has a melting point of about −10 °C, so it is liquid under the conditions of the simulations.

The nonbonded interactions (i.e., the DPD conservative force) are defined by standard DPD pairwise soft repulsions of the form^28,29^
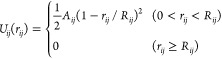
4where *A*_*ij*_ and *R*_*ij*_ are the repulsion amplitude and range, and  is the separation between beads *i* and *j* located at  and  respectively. The repulsion amplitude for water beads is set to *A*_*ij*_ = 25, and likewise by convention *R*_*ij*_ = 1 for water beads, in units of *r*_c_, the fundamental unit of length in our model.

We establish the latter by mapping our bead model of pure water at a bead density *ρr*_c_^3^ = 3 to real water using Avogadro’s constant *N*_A_, the molar mass of a water bead *M*(2H_2_O) ≃ 36 g mol^–1^ (noting there are two water molecules per bead), and the mass density of water ϱ_w_ ≃ 1000 g L^–1^. From this we infer that
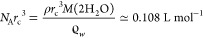
5and hence *r*_c_ ≃ 0.564 nm. Note that with eq 5 we can convert a bead density in DPD units to a real density by multiplying by *M*(bead)/*N*_A_*r*_c_^3^, where *M*(bead) is the molar mass.

The remaining bead parameters are given in Table 1 and were tuned by Anderson et al.^1^ to reproduce the experimental liquid phase densities and water–octanol partition coefficients (log *P* values) for a range of small molecules including, relevant for the present study, dodecane. For the bonded interactions, a simple harmonic potential *U*_*ij*_^B^ =  is used between connected DPD beads, with *r*_0_ equal to 0.29 and 0.39 for CH_3_– CH_2_CH_2_ and CH_2_CH_2_– CH_2_CH_2_ respectively, and a single bond constant *k*_b_ = 150 (DPD units) adopted throughout. This ensures the bond lengths approximately match the distance between the coarse-grained chemical groups.^1^ Bond rigidity is enhanced by adopting the three-body angular potential used by Venturoli et al.,^30,31^ namely, *U*_*ijk*_^A^ =  where θ is the angle between adjoining bonds, using θ_0_ = 180° and *k*_*a*_ = 5 (DPD units) for all angles. With these choices, the coarse-grained molecular configurations are compatible with the underlying molecular architectures, given the above length scale mapping. The cutoff distance for the dissipative and random forces required in the DPD method was assigned equal to the maximum cutoff distance in the system (i.e., max ({*R*_*ij*_}) = 1.0740) and the dissipative friction amplitude was set at 4.5.

**Table 1:** DPD Repulsion Amplitudes and Cut-Off Distances for the Three Bead Types Used in This Work Obtained from Anderson et al.^1^

bead *i*	bead *j*	*A*_*ij*_	*R*_*ij*_
2H_2_O	2H_2_O	25.0	1.0000
2H_2_O	CH_2_CH_2_	45.0	1.0370
2H_2_O	CH_3_	45.0	0.9775
CH_2_CH_2_	CH_2_CH_2_	22.0	1.0740
CH_2_CH_2_	CH_3_	23.0	1.0145
CH_3_	CH_3_	24.0	0.9550

### Wall Model

We now turn to the specification of the wall model. As mentioned, in our approach we treat this as a smooth, featureless substrate, which interacts with the DPD beads through bead-specific wall potentials. For instance, consider a wall located at the *z* = 0 plane. We suppose generally there is a hard reflecting barrier at *z* = 0, so that DPD beads cannot penetrate to *z* < 0. This is implemented in the simulation using specular (mirror-like) reflection (rather than bounce-back reflections). For clarity in a specular reflection scheme, where a DPD bead passes the plane of the wall in a given time step, the final position of a DPD bead is updated to account for the change in sign of the velocity vector perpendicular to the plane of the wall as the bead passes the plane. For *z* > 0 we augment this with a soft, short-range potential of the form^32^
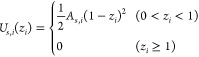
6where *z*_*i*_ is the height of the *i*-th DPD bead above the hard reflecting boundary at *z* = 0. We set the wall potential to have the same functional form for all bead types but differentiate these from each other by bead-specific repulsion amplitudes *A*_s,*i*_, intended to capture the surface chemistry. For simplicity, we set the range here equal to unity (in units of *r*_c_) for all bead types though one could envisage this being used as an additional fine-tuning parameter. We anticipate that the exact functional form of the wall potential is unimportant as long as it remains soft and short-ranged, so we have chosen this form for numerical convenience, which is similar to the pair interactions in eq 4.

Again for simplicity, in the present study we do not distinguish between the two alkane bead types, so we only consider wall repulsion amplitudes *A*_s,dod_ where “dod” (for dodecane) represents both CH_3_ and CH_2_CH_2_ beads, and *A*_s,wat_ where “wat” refers to the water beads. These repulsion amplitudes were varied from zero to 50 in steps of 10 (in a matrix) to observe the effect of the interaction strength upon the structure and surface energies for each of the two liquids, water and dodecane.

### Simulation Conditions

#### General Simulation Conditions

All simulations were run using the dl_meso simulation package (version 2.8),^33^ at a reduced temperature of *T* = 1.0 (which is equivalent to 25 °C according to the underlying parametrization). A time step of 0.02 DPD units was used throughout. For *NPT* simulations a Langevin-piston barostat was used, with *p* = 23.7 in reduced units to correspond to the pressure in our bead model of pure water at a reduced density *ρr*_c_^3^ = 3.

We note that since *k*_B_*T*/*r*_c_^3^ = *RT*/*N*_A_*r*_c_^3^ ≃ 2.3 × 10^7^ Pa at *T* = 298 K, in real units the operating pressure 23.7*k*_B_*T*/*r*_c_^3^ ≃ 0.54 GPa. This is much larger than the atmospheric pressure (100 kPa). We accept this, noting that unlike typical real liquids, there is no cohesive contribution to the forces between DPD beads to reduce the “overpressure”.

We have found that at *P* = 23.7 the reduced density of pure DPD dodecane is *ρr*_c_^3^ ≃ 3.27. Since in this DPD model dodecane is a 7-mer, the mean molar mass of a dodecane bead is *M*(C_12_H_26_)/7 ≃ 24.3 g mol^–1^. Utilizing eq 5, this corresponds to a real mass density 3.27 × 24.3/0.108 ≃ 740 g L^–1^, which is within 1.5% of the true value of 750 g L^–1^. This is expected since the DPD parameters are tuned to reproduce liquid phase densities.

#### Liquid–Liquid Interface

Each system utilized an initial simulation cell of size *L*_*x*_ × *L*_*y*_ × *L*_*z*_ = 10 × 10 × 200. This size was settled upon after performing calculations for a series of different box sizes to ensure that finite size effects are ruled out. Further information regarding these effects can be found in the Supporting Information. For the dodecane–water system, the initial simulation setup was constructed such that each of the two components was placed in either half of the simulation cell. Periodic boundary conditions were adopted in all three dimensions. In this case, the oil–water system comprises two interfaces: one at *z* ≈ *L*_*z*_/2 and one at *z* ≈ 0. In the final result, while equal in number of beads, water and dodecane occupy slightly different volumes due to their different densities. All simulations to determine the interfacial tension values were performed initially under an *NPT* ensemble at starting reduced density of ρ = 3.0. Subsequently, *NVT* simulations were completed (at the equilibrium simulation box lengths from *NPT* calculations) to ensure agreement in results. Both ensembles produced results in agreement within the reported error of each other.

For comparison and to provide a benchmark, additional molecular dynamics (MD) simulations were performed at a temperature of 300 K using the TIP4P/2005 water model^34^ and dodecane obtained using the optimized L-OPLS parameters of Siu et al.^35^ A system containing 1600 dodecane and 20 500 water molecules (corresponding to a final box size of *L*_*y*_ = *L*_*z*_ = 8.5 nm and *L*_*x*_ = 17.1 nm) was initially run for 5 ns (using a time step equal to 0.001 ps) under the semi-isotropic *NP*_*zz*_*T* ensemble, in which the *L*_*x*_ and *L*_*y*_ dimensions are fixed and *L*_*z*_ fluctuates to reach the pressure of 1 bar, using the Parrinello–Rahman barostat and Nosé–Hoover thermostat. The system was further run at *NVT* conditions for 20 ns and the final 10 ns were used for data collection.

#### Liquid–Surface Interface

For the liquid–surface systems, molecules comprising the liquid were placed randomly in a simulation cell equivalent in size to the dodecane–water system, defined above. The same simulation protocol as above was used except that the semiperiodic boundary conditions were employed in the *xy* dimensions and substrate walls are placed at *z* = 0 and *z* = *L*_*z*_. The properties of the upper wall are identical to those of the lower wall, with suitable reflection and translation.

#### Droplet on Surface

To validate the Young equation, we also undertook explicit droplet simulations, using the same surface as described above, parallel to the *xy*-plane, and *NVT* conditions. For this problem, the use of cylindrical droplets provides some technical advantages: the effects of a possible line tension on a curved contact line are eliminated, the droplet shape is easier to analyze (by projecting along the droplet axis), and the profile more sharply defined because a larger number of DPD beads are involved. In fact, we tested both cylindrical and spherical droplets and found that spherical droplets were indeed more difficult to work with, in particular when dealing with small systems and small contact angles. We can also remark that in a simulation, in contrast to experiment,^36^ cylindrical droplets (provided they are not too long) are stabilized against a Rayleigh-Plateau instability by the periodic boundary conditions.

**Figure 2:**
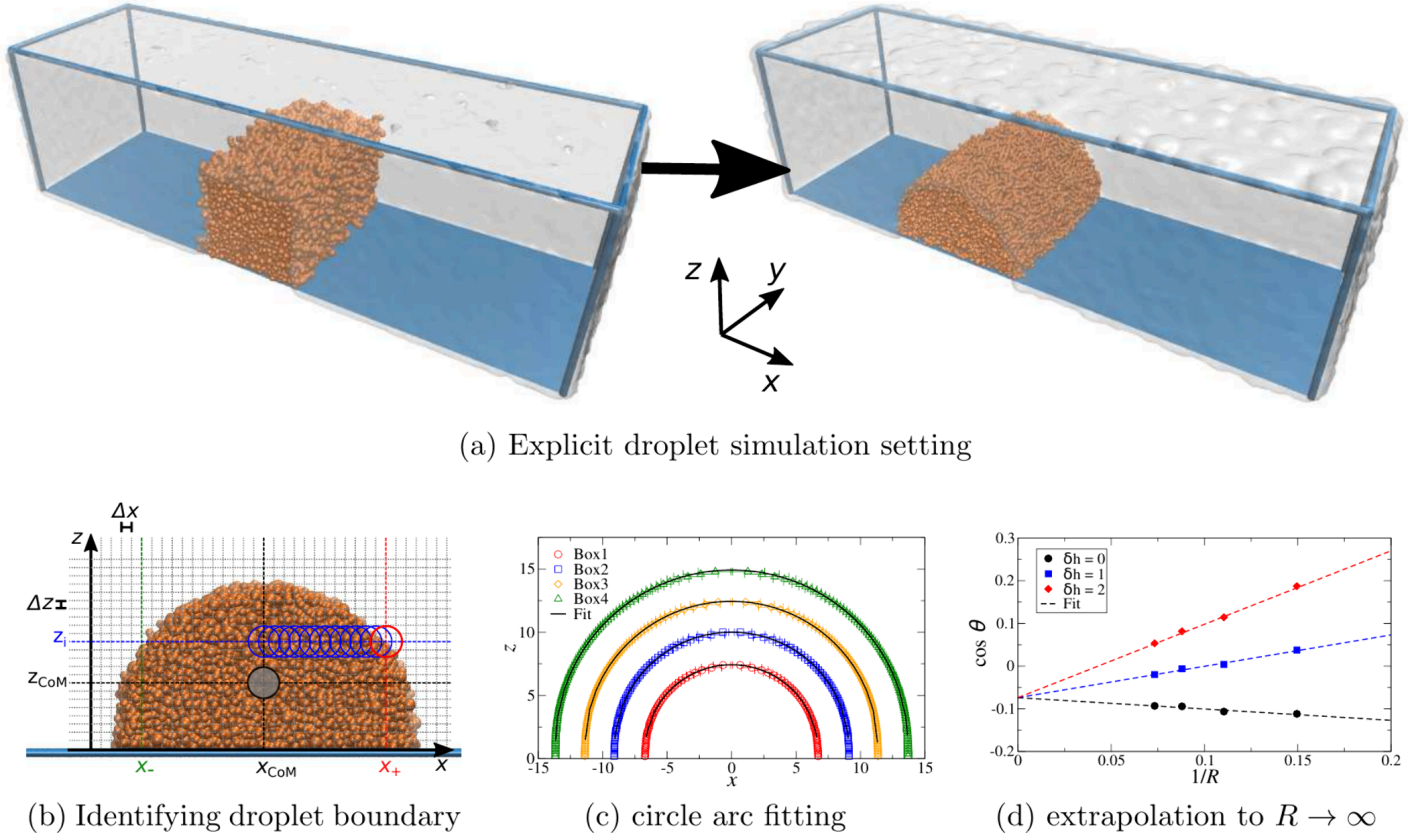
Stages required to measure cos θ_sim_ in explicit droplet simulations. Here we show (a) the initial and final simulation configuration, were water beads are represented as a transparent surface for clarity, (b) a schematic representation of the algorithm to obtain the discrete droplet profile in the *xz* plane, (c) the circle-arc profile from systems of different box sizes using *A*_s,dod_ = *A*_s,wat_ = 50, and (d) a demonstration of the interface position *δh* effect on the extrapolation to infinite droplet radius using the same data in (c).

The initial configuration consisted of dodecane molecules within a rectangular parallelepiped region at the reduced dodecane bead density of ρ = 3.27 in DPD units, located on the surface at *z* = 0, centered in *x* and extending along the *y* axis. The rest of the simulation box is filled with DPD water beads at a reduced density ρ = 3. The visualization of an initial configuration is presented in Figure 2a. The distance between the walls *L*_*z*_ is large enough to avoid interactions between the dodecane molecules and the upper surface. We select interaction parameters *A*_s,α_ to produce a variety of contact angles in the explicit systems. The simulations are equilibrated for 5 × 10^5^ time steps to allow the dodecane region to adopt a cylindrical shape (see Figure 2a, right-hand panel). Once equilibrium is reached, production simulations of 10^6^ time steps are performed and a configuration is saved every 10^3^ time steps for analysis.

For every surface model, we studied four simulation boxes to produce droplets of different diameters. In Table 2, we give size and composition details: total dimensions (*L*_*x*_, *L*_*y*_, *L*_*z*_), the size of the initial dodecane region (*l*_*x*_, *l*_*y*_, *l*_*z*_), the number of dodecane molecules *N*_dod_ (DPD 7-mers), the number of DPD water beads *N*_wat_ = *N*_H_2_O_/2, and the total number of DPD beads in every box *N*_tot_ = 7*N*_dod_ + *N*_wat_.

**Table 2:** Box Details for the Simulation of Explicit Dodecane Droplets in Water^a^

Box ID	(*L*_*x*_, *L*_*y*_, *L*_*z*_)	(*l*_*x*_, *l*_*y*_, *l*_*z*_)	*N*_dod_	*N*_wat_	*N*_tot_
Box1	(50, 20, 25)	(8, 25, 8)	907	69 164	75 513
Box2	(50, 20, 25)	(11, 25, 11)	1679	64 197	75 950
Box3	(100, 30, 30)	(14, 30, 14)	3115	249 958	271 763
Box4	(100, 30, 30)	(17, 30, 17)	4512	240 970	272 554

a The box size (*L*_*x*_, *L*_*y*_, *L*_*z*_) and size of the dodecane regions (*l*_*x*_, *l*_*y*_, *l*_*z*_) are given in DPD units. The final columns report the number of dodecane molecules (DPD 7-mers), the number of DPD water beads, and the total number of DPD beads.

From the balance between viscous dissipation and surface tension, the time for the droplet to reach its final shape can be estimated as *ηR*/γ, where η is the fluid viscosity, γ is the interfacial tension, and *R* is the droplet diameter.^22^ Since we expect η and γ to be *O*(1) in DPD units, and *R* is at most several tens of *r*_c_ (Table 2), this time scale is perhaps 10–100 DPD time units, or not more than 10^3^–10^4^ DPD time steps. Hence the equilibration time of 5 × 10^5^ time steps should be ample to allow the droplet to reach its final cylindrical shape. This is in accordance with our observations.

### Measurement

#### Liquid–Liquid Interfacial Tension Determination

The approach to calculating the liquid–liquid interfacial tension is well established,^37^ and uses the volume- and time-averaged diagonal components of the pressure tensor, ⟨*P*_*xx*_⟩, ⟨*P*_*yy*_⟩, and ⟨*P*_*zz*_⟩, as
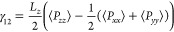
7Here *L*_*z*_ is the length of the simulation box in the direction perpendicular to the liquid–liquid interface, assumed to be the *z*-direction and the outer factor “2” derives from the fact that in periodic boundary conditions there are two interfaces. In a bulk region, all three components of the pressure tensor become equal to each other and equal to the bulk pressure, *P*_*xx*_ = *P*_*yy*_ = *P*_*zz*_ = *p*, and the only criterion for *L*_*z*_ is that it be sufficiently large for the simulation box to contain two well-separated interfaces. A nonzero value for γ_12_ (which must be positive) results from the fact that *P*_*xx*_ = *P*_*yy*_ (assuming in-plane isotropy) deviates negatively from *P*_*zz*_ = *p* in the interfacial region, where the constancy of the latter derives from mechanical equilibrium. Typically, the negative deviation in the lateral components of the pressure tensor corresponds to a “dip” in the density profiles in the interface, with fewer DPD beads, reduced lateral forces, and reduced lateral momentum transport in this region.

#### Liquid–Substrate Surface Energy Determination

In contrast, determination of the surface energy for a fluid against a wall can be fraught with difficulties due to the presence of internal stresses in the solid substrate. To overcome these, Leroy and co-workers devised a “phantom wall” method to compute the surface energy.^38−40^ By incrementally “lifting” the phantom wall off the real substrate, one can compute the difference in surface energy between the real wall and the phantom wall by thermodynamic integration. This is augmented by a direct calculation of the phantom wall surface energy once the real wall is sufficiently distant to avoid perturbation of the fluid.

In our model, the introduction of a phantom wall and the thermodynamic integration step are unnecessary since the wall is structureless, there are no internal stresses, and the wall forces are always normal to the surface. As a result, we can show that eq 7 can also be used for the wall surface energies, provided an augmented definition  is used which includes the wall forces. The details are given in section S1 of the Supporting Information.

Importantly, the surface energy thus computed can be positive *or negative*. The reason is that there does not have to be a diminishment in *P*_*xx*_ = *P*_*zz*_ at the wall. In fact, the DPD bead density profile can show an excess, for example, for a pure hard wall without any additional repulsive force. In such a situation DPD beads pile up in the vicinity of the wall because a part of the *transverse* repulsions between beads are missing, as a consequence of beads being excluded from the *z* < 0 and *z* > *L*_*z*_ regions.

During the simulation we measure the pressure tensor every 50 time steps, obtained as output of dl_meso, and incorporating the contribution of the wall forces as described in section S1 of the Supporting Information. This is the default position in dl_meso 2.8 onward but not for earlier versions of the package.

#### Droplet Contact Angle

To obtain the contact angles from an explicit droplet, we perform the following steps based on similar protocols by Tenney and Cygan^41^ and Kanduč.^42^ First, we identify the droplet boundary by following the schematic representation in Figure 2b. We start by locating the droplet center of mass (CoM) onto the *xy* plane. The local density of a cylindrical cell centered at the CoM is stored as a reference value ρ_ref_ = ρ(*x*_CoM_, *z*_CoM_), using a cell radius of *R*_cell_ = 1.5. The local dodecane densities are calculated by moving the center of the sampling cell on a grid with Δ*x* = Δ*z* = 0.2, starting from the base of the droplet (by the surface) to the droplet’s apex. The discrete profile boundary at height *z*_*i*_ is determined as the position of the first cell to the left (*x*_–_(*z*_*i*_)) and right (*x*_+_(*z*_*i*_)) from *x*_CoM_, which satisfies ρ(*x*_*k*_, *z*_*i*_)/ρ_ref_ < 0.5.

With the droplet boundaries defined, a circular arc can be fitted around the perimeter. The profile points close to the top of the drop present larger error bars due to the density fluctuations in that region (Figure 2); hence, we discard some of those profile points with the criterion of using only data with less than 10% of relative error. For the remaining data, we use the algorithm presented in Thomas and Chan^43^ based on the Landau method to obtain the radius *R* and center (*x*_*c*_, *z*_*c*_) of the circle arc.

The contact angle θ_sim_(*R*) is obtained from
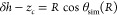
8where *δh* is the interface position. Finally, for every set of parameters, we exploit the linear dependence of *R* in eq 8 to extrapolate out to *R* → *∞* and obtain θ_sim_ ≡ θ_sim_(*R* → *∞*). We present a test of the sensitivity to *δh* in Figure 2d using systems with *A*_s,wat_ ≡ *A*_s,dod_ = 50. We observe that while the individual cos(θ_sim_) values are affected by the *δh* selection, the dependence is lost in the macroscopic droplet extrapolation, *R* → *∞*. This is compatible with the observations in Zhang et al.,^44^ where comprehensive discussion on the topic is presented. The final analysis of all simulation sets is performed using *δh* = 2 in DPD units. We note that by comparing cylindrical and spherical droplets, the further effects of contact line tension on the latter could be isolated. This is not something we have attempted here.

## Results and Discussion

### Interfacial Tension and Surface Energies

In this section, we use DPD simulations to determine the interfacial tension and surface energies that are required in the Young equation, eq 1, using the volume-average pressure tensor components in eq 7 as detailed above. We convert the results from DPD units to real units by multiplying by the factor *k*_B_*T*/*r*_c_^2^ = *r*_c_ × *RT*/*N*_A_*r*_c_^3^ ≃ 12.9 mN m^–1^, on the basis of eq 5 and assuming *T* = 298 K.

From simulations of the dodecane/water system (without walls), we calculate the dodecane/water interfacial tension to be γ_wat,dod_ = 27.3 ± 0.4 mN m^–1^. Despite the large background “overpressure” (see simulation conditions in the Materials and Methods), this is comparable to, but somewhat lower than, the experimentally reported 51–52 mN m^–1^ range that is typical for this system.^45,46^ We attribute the mismatch between our simulated results and experimental data to a rather thick liquid–liquid interfacial region (solid lines in Figure 4a), which is a consequence of the soft potentials typically employed in DPD. This is discussed further in the Conclusions of this article

**Figure 3:**
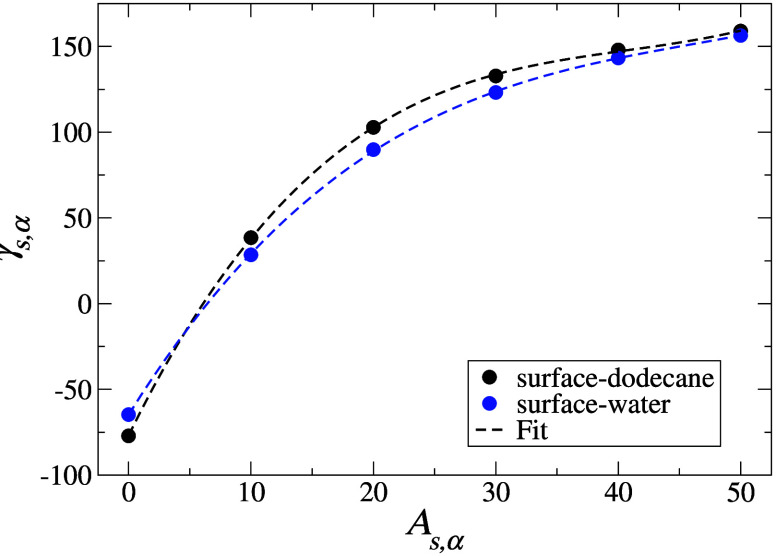
Surface energies in mN m^–1^ for dodecane and water using different surface–fluid interactions (circles), together with fits to third-degree polynomials (dashed lines).

**Figure 4:**
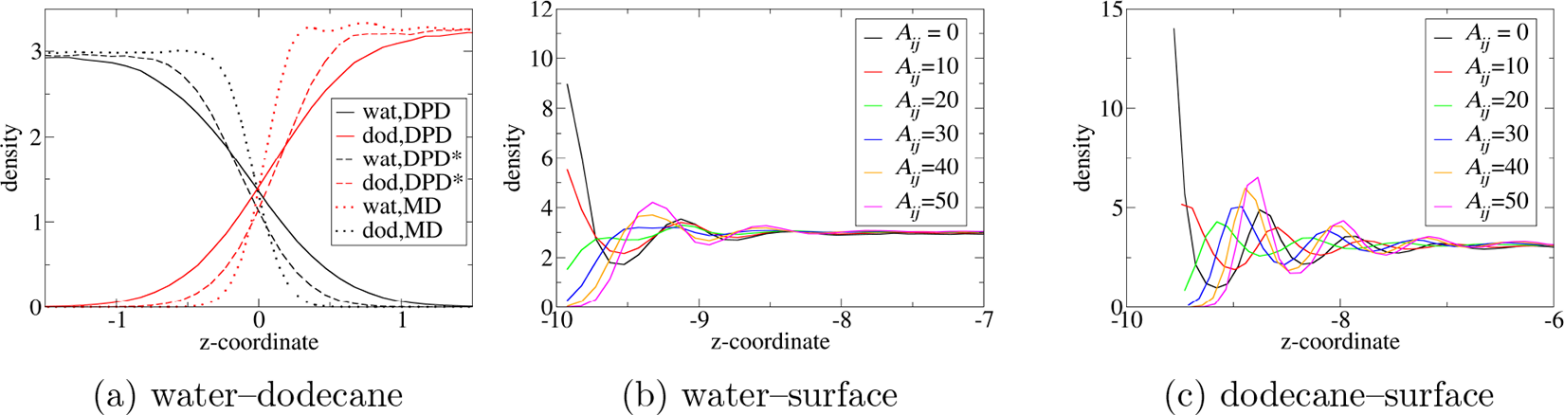
Liquid bead number concentration profiles perpendicular to the interface, water phase giving way to dodecane phase in (a) and with surface placed at lower *z* boundary in the case of (b, c). For (a) we show the water–dodecane interface for three scenarios: (i) resulting from the original DPD parameters (solid lines); (ii) resulting from an increase in the water–dodecane repulsion parameter (from 45 to 100, denoted by an * in the legend); (iii) resulting from MD simulations. For details of the simulations carried out, please refer to the main text and the discussion in the conclusion section. For (b) and (c) we show the concentration profile for each liquid with the surface at varying *A*_*ij*_ values. Data were extracted from simulation trajectories using the ummap analysis code.^47^

Now we turn to the substrate surface energies, using simulations of either pure water or pure dodecane, confined between parallel walls as described in the section where liquid–liquid interfacial tension determination is discussed in Materials and Methods. Table 3 and Figure 3 shows how the surface energies for water and dodecane vary with the surface repulsion amplitude, *A*_s,α_ (where α is either dodecane or water). For both liquids, the value of γ_s,α_ increases strongly, as expected. In the case where the interaction between the surface and the liquids is set to zero, the recorded surface energies are large and negative (γ_s,wat_ = −64.7 mN m^–1^ and γ_s,dod_ = −77.1 mN m^–1^). Figure 4 panels b and c present the density profiles of the water and dodecane liquids at the surface with increasing *A*_s,α_. For both liquids, significant ordering results, with dodecane being more strongly affected than water. Ordering in the case of water persists for ≈2 DPD distance units, and for dodecane this is increased to ≈3 units. Surface induced ordering is minimized for both liquids at *A*_s,α_ ≈ 20.

**Table 3:** Substrate Surface Energies, in mN m^–1^, for Various Wall Interaction Strengths^a^

*A*_s,α_	γ_s,dod_	γ_s,wat_
0	–77.1(5)	–64.7(4)
10	38.5(5)	28.5(4)
20	102.8(5)	89.8(4)
30	132.8(5)	123.1(4)
40	147.9(5)	143.3(4)
50	158.9(5)	156.4(4)

a α represents any bead species in water and dodecane. Results correspond to values obtained for a cell of size *L*_*x*_ × *L*_*y*_ × *L*_*z*_ = 10 × 10 × 200. The figure in brackets is an estimate of the error in the final digit.

### Validation of Contact Angle Prediction

**Figure 5:**
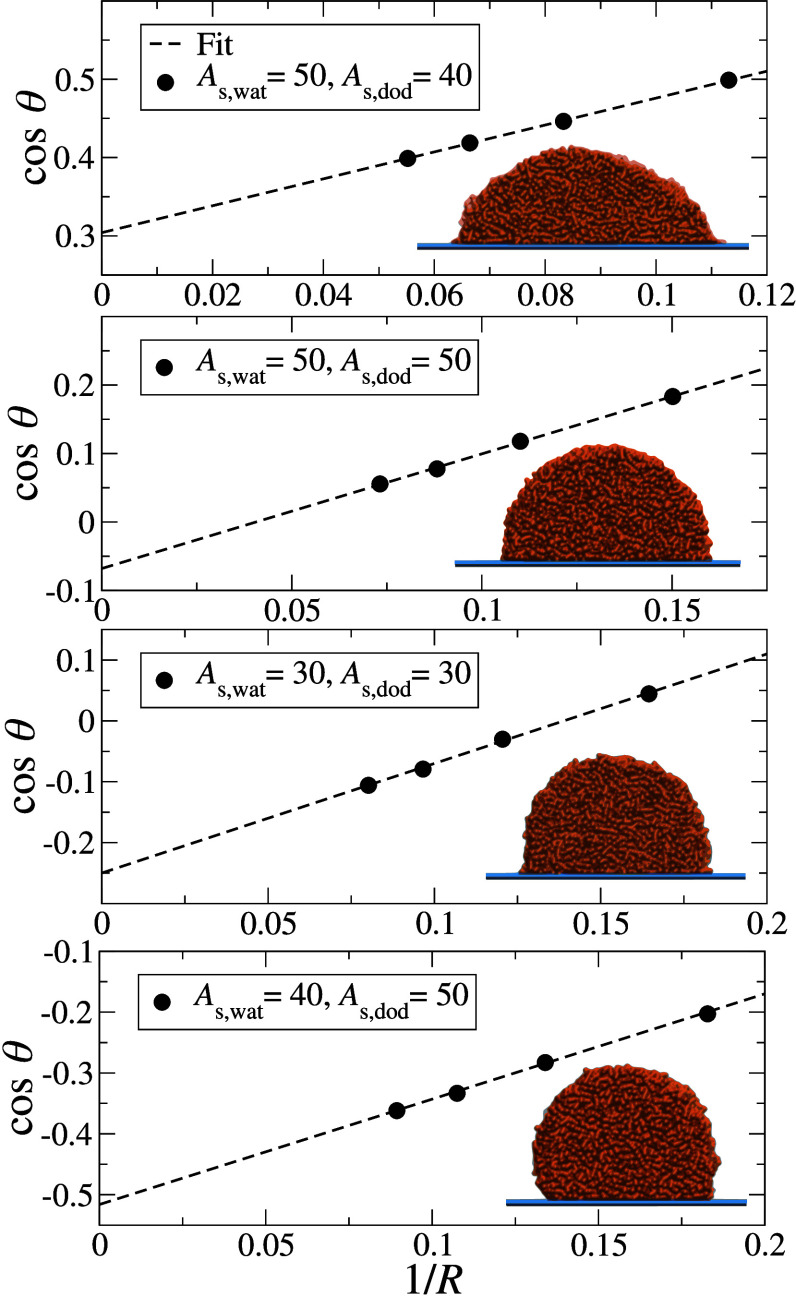
cos θ_sim_(*R*) values obtained from explicit droplet simulations of different sizes. The dashed lines represent the linear extrapolation to the macroscopic radius, *R*^–1^ → 0. Inserted in the plot, a visualization of the largest system is shown for every pair of wall–fluid interactions.

We now validate our approach by comparing the contact angles predicted by the Young equation to measurements of the contact angles from our explicit droplet simulations. For the latter, examples of the cos θ_sim_(*R*) values and linear extrapolations are presented in Figure 5 for selected cases. We include snapshots of the largest droplet in equilibrium for every case and omit the error bars, which are smaller than the symbols.

**Figure 6:**
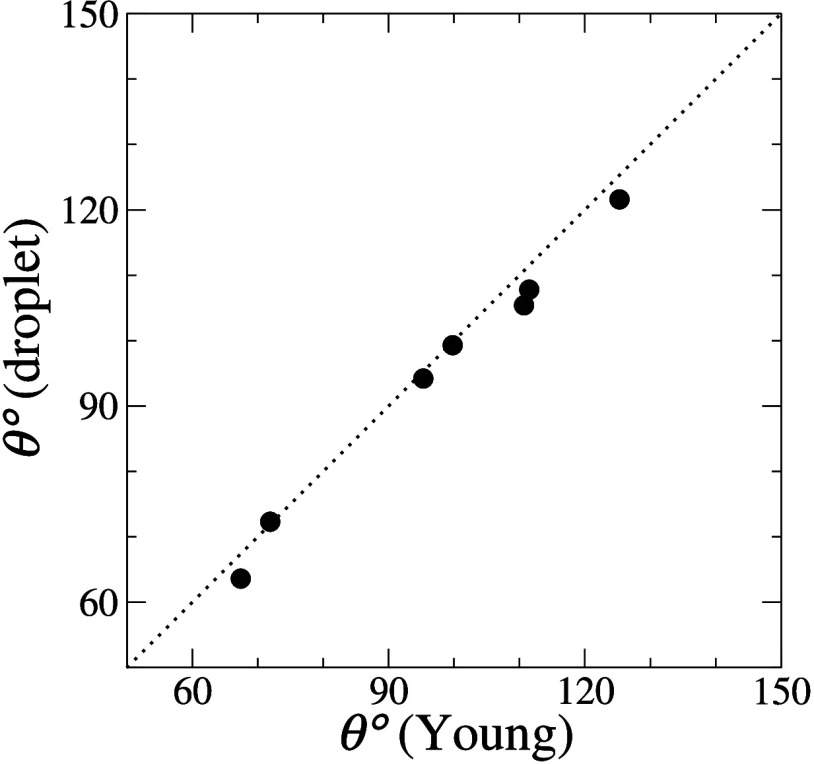
Contact angles measured in droplet simulations (vertical axis) plotted against the values predicted by the Young equation (horizontal axis).

Table 4 shows the comparison between the contact angles predicted from the Young equation using the water/dodecane interfacial tension γ_wat,dod_ = 27.3 ± 0.4 mN m^–1^ (i.e., using our original parameter set in Table 1) plus the surface energies reported in Table 3, and the contact angles extrapolated from explicit droplet simulations by the described method, for a variety of *A*_s,α_ combinations. A good agreement is found with a relative error under 4%. In Figure 6, we show a scatter plot of the data.

**Table 4:** Comparison of Contact Angles Obtained via the Young Equation and Explicit Droplet Simulations^a^

*A*_s,wat_	*A*_s,dod_	θ [deg] (Young)	θ [deg] (droplet)
10	10	111(4)	108(3)
30	30	111(3)	105(3)
30	40	155(2)	141(5)
40	30	67(3)	64(2)
40	40	100(2)	99(3)
40	50	125(3)	122(4)
50	40	72(2)	72(2)
50	50	95(2)	94(3)

a The figure in parentheses is an estimate of the error in the final digit.

To extend the tests to other surface–fluid interactions, we fit the discrete values of γ_s,α_ (in real units of mN m^–1^) as a function of *A*_s,α_ to third-order polynomials,
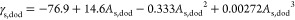
9a
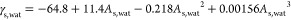
9bThe coefficient of determination was *R*^2^ = 0.9999 in both cases, since the dependence is rather smooth and these fits are quite accurate, as shown in Figure 3. The coefficients in these expressions differ because dodecane in our DPD model is a molecular fluid at a different density and with different bead interactions than the DPD water model.

**Figure 7:**
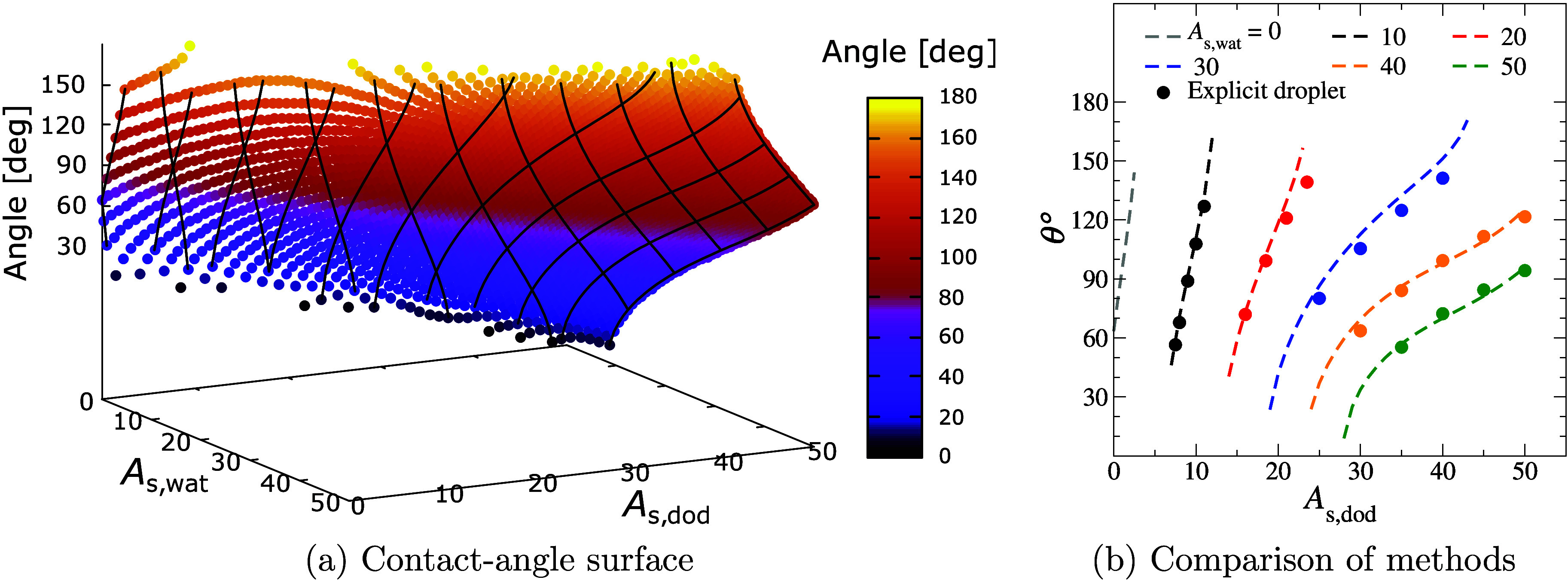
Contact angles obtained from the Young equation. (a) Surface representation using smooth values of γ_s,wat_ and γ_s,dod_ and (b) 2D representation using the smooth γ_s,dod_ in the Young equation (dashed lines) and comparing it to explicit droplet simulations (filled circles).

These fits allow us to represent the contact angles from the Young equation as a surface; see Figure 7a. Using the discrete γ_s,wat_ values, 2D projections of the contact angle as a continuous function of *A*_s,dod_ are shown in Figure 7b, with dashed lines. We employ this plot as a guide to select (*A*_s,wat_, *A*_s,dod_) combinations and simulate the explicit droplet systems. The resulting contact angles correspond to the solid symbols in Figure 7b. We observe a good agreement finding an average relative error of 5% when comparing both approaches.

**Figure 8:**
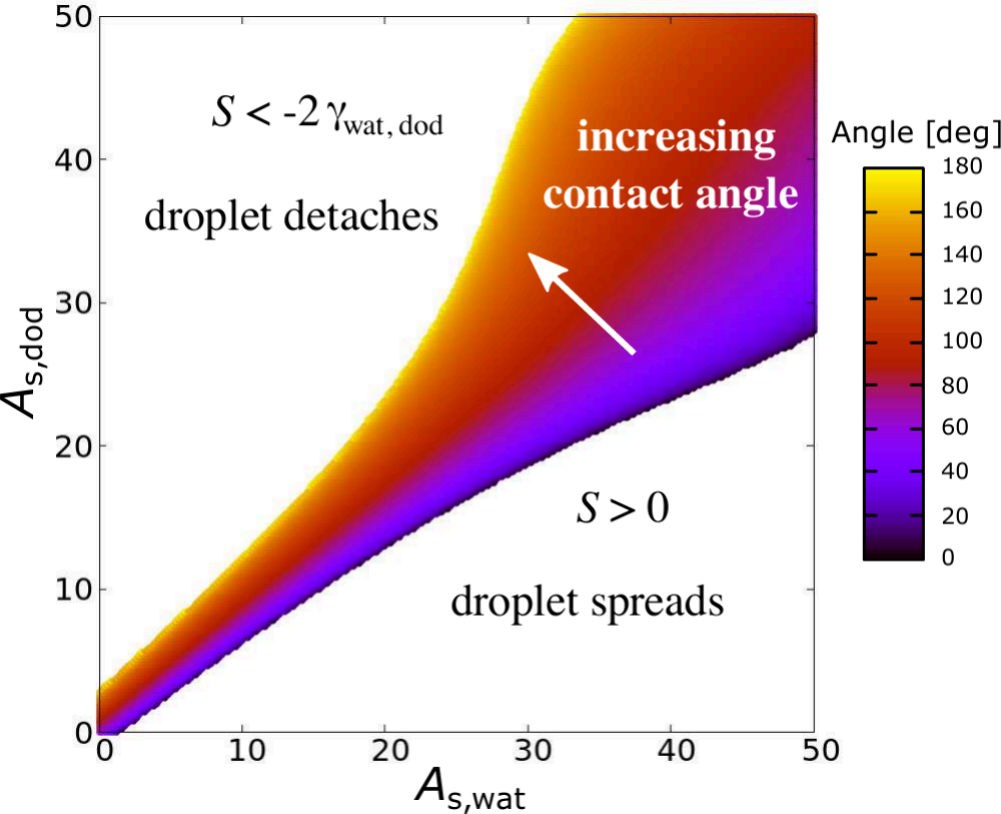
Map of droplet behaviors on the surface as a function of (*A*_s,wat_, *A*_s,dod_), as determined by the de Gennes spreading parameter *S*.

Combinations of (*A*_s,wat_, *A*_s,dod_) that produced contact angles under 30° were challenging to simulate since the explicit droplets tend to spread out, in particular, for the smaller system sizes. Other combinations generated detached droplets that we did not analyze. To understand these behaviors, we return to the de Gennes spreading parameter defined in eq 2. Here, we use the smoothed versions of surface energies in eq 9 to calculate *S* as a function of (*A*_s,wat_, *A*_s,dod_), noting that for this purpose γ_wat,dod_ = 27.3 ± 0.4 mN m^–1^ is a constant dictated by the coarse-grained water-plus-dodecane model. Figure 8 shows the behavior of the droplet on the surface as predicted by *S*. Corresponding to the classification scheme in the introduction, three regimes are observed. In the lower right region, *S* > 0 and the droplet spreads out. In the upper left region, *S* < −2γ_wat,dod_ corresponds to droplet detachment. In the middle region, where −2γ_wat,dod_ < *S* < 0, partial wetting is observed, with the droplet sitting on the surface with a finite contact angle as indicated by the color scale. The boundary between the droplet spreading regime and finite contact angle regime corresponds to the boundary we have seen in the simulations.

These behaviors follow common sense, since increasing *A*_s,α_ increases the surface energy of the corresponding phase. Thus, generally, the surface tends to favor the phase with the smaller value of *A*_s,α_. Finally, we note that the map in Figure 8 is asymmetric for the same reason that the polynomial fit coefficients in eq 9 are different: in our model water and dodecane are different liquids with different densities and different bead interactions.

### Application: Tuning the Hydrophobicity of Model Surfaces

In this section, we employ the Young equation to parametrize surfaces with different degrees of hydrophobicity. As a benchmark, we use experimental contact angles of water droplets in dodecane in silica surfaces obtained by Andersson et al.^48^ In these experiments, silica wafers are modified with self-assembled monolayers (SAMs) of different compositions, affecting the wettability. Here, we take three combinations of −OH and −CH_3_ coverages (see first and second columns in Table 6) and propose a simple continuous wall model for each.

**Figure 9:**
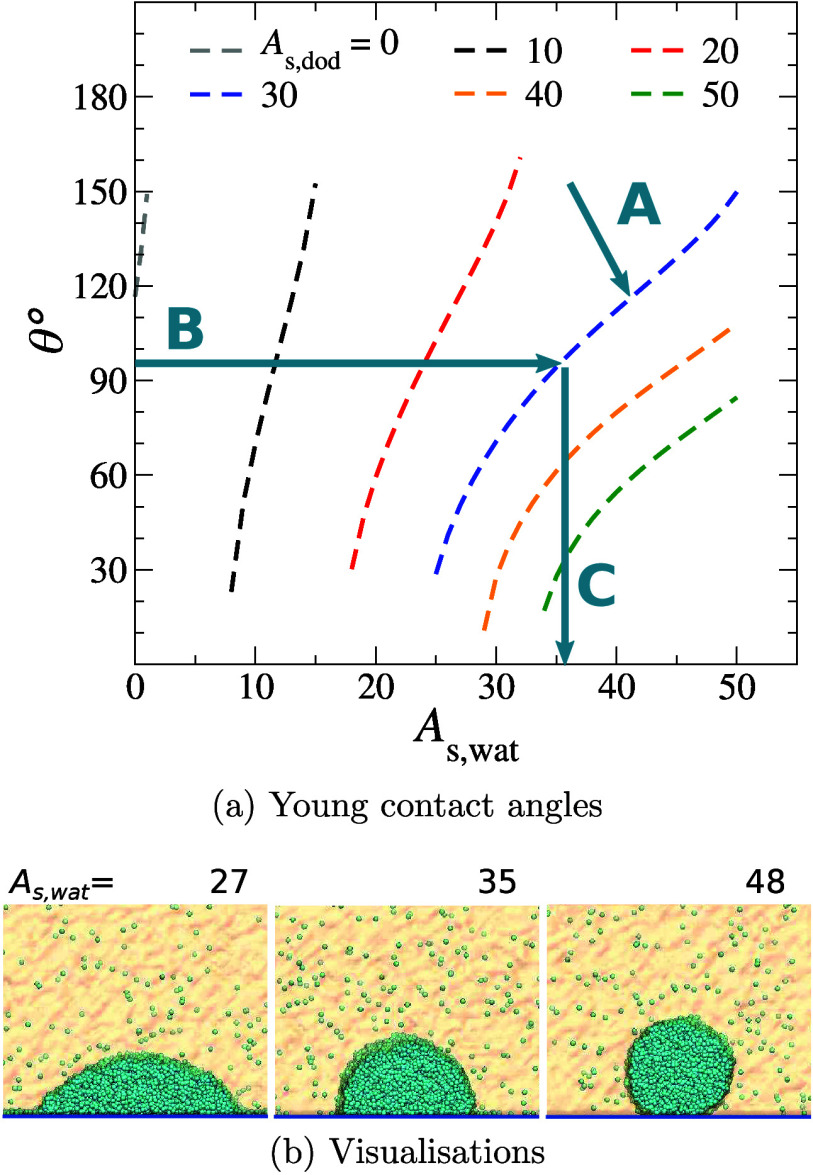
(a) Contact angles from the Young equation for a DPD water droplet in dodecane. The arrows indicate the steps followed in the surface parametrization. (b) Snapshots of explicit droplet simulations using model surfaces with different hydrophobicity degrees.

We begin with eq 1, where the droplet is now made of water and the surrounding liquid dodecane. The two-phase simulations required to obtain the interfacial tension for the Young equation are the same cases presented in the Results and Discussion, where interfacial tension and surface energies are discussed. In analogy to the previous section, we use the discrete γ_s,dod_ values and the smooth version of γ_s,wat_, eq 9b, to plot the contact angles from the Young equation as a continuous function of *A*_s,wat_, Figure 9a. To generate continuous wall models with effective interactions, we follow the steps represented in the same figure: A) select and fix *A*_s,dod_ = 30, leaving *A*_s,wat_ to reflect the hydrophilic or hydrophobic effect introduced by the surface modifications; B) locate the experimental contact angles in the curve and C) find the corresponding *A*_s,wat_ value. The effective surface water interactions for the cases selected are given in the third column of Table 6.

Finally, we confirm that the surface parametrization reproduces the target contact angles using explicit droplet simulations. A complete list of the simulated systems is given in Table 5, and the analysis method is applied as described in the simulation conditions section of Materials and Methods. The droplets are shown in Figure 9b, and the measured contact angle values are presented in the last column of Table 6. The results are in good agreement with the experimental values (where the average standard deviation is 6°), with relative errors between 2 and 10%. Physically, the surface in the model has a higher hydrophobicity as the repulsion amplitude *A*_s,wat_ increases.

**Table 5:** Box Details for the Simulation of Explicit Water Droplets in Dodecane^a^

Box ID	(*L*_*x*_, *L*_*y*_, *L*_*z*_)	(*l*_*x*_, *l*_*y*_, *l*_*z*_)	*N*_dod_	*N*_wat_	*N*_tot_
Box1	(78, 26, 32.5)	(13, 26, 13)	28 648	13 183	213 719
Box2	(90, 30, 37.5)	(15, 30, 15)	44 010	20 251	328 321
Box3	(102, 34, 42.5)	(17, 34, 17)	64 065	29 479	477 934
Box4	(114, 38, 47.5)	(19, 38, 19)	89 441	41 155	667 242

a For column details see Table 2.

**Table 6:** Experimental Contact Angles from Andersson et al.^48^ and Simulated Values in This Work, for SAMs of Different Quality^a^

SAM composition		Exp θ [deg]	*A*_s,wat_	Simul θ [deg]
72% – OH	28% – CH_3_	51	27	56(2)
48% – OH	52% – CH_3_	95	35	97(2)
22% – OH	78% – CH_3_	139	48	142(5)

a The figure in brackets is an estimate of the error in the final digit.

## Conclusions

In this work, we set out to develop the basis for the development of chemically specific surface interactions for dissipative particle dynamics simulation. We compared explicit droplet simulations with independent computations of the contact angle from the Young equation using separate measurements of the interfacial tension and surface energies, finding good agreement. We then demonstrated how our model can be used to parametrize a SAM-covered silica surface to obtain a given contact angle for a water droplet in dodecane sitting on the surface. Figure 9a is the key result here, since it shows how to choose the wall interaction parameters to obtain a specified contact angle in this particular system, but obviously, the method can be applied to other systems. Our results show that surfaces can be easily and rapidly parametrized via the surface energies, which are computable with a relatively trivial extension of the pressure tensor method for computing the interfacial tension between liquids. Realistic surfaces can be modeled by tuning the interactions between DPD beads and the wall, using experimental contact angles as a benchmark, without having to undertake expensive explicit droplet simulations for calibration. We have focused on flat, structureless surfaces, but in principle the methods can be extended to structured surfaces, although the computation of the surface energies becomes complicated if one has to implement a “phantom wall” and lift it off the surface, in a thermodynamic integration step.^38−40^

As already noted, the DPD force field that we have used for this study underpredicts the dodecane/water interfacial tension by about 45% (i.e., 27.3 ± 0.4 mN m^–1^ in the model; compared to ≃50 mN m^–1^ in reality). A number of DPD studies report models where the interfacial tension for oil–water systems is closer to experimental data. In these works, both oil and water are often represented as single beads and the repulsion amplitude describing the interaction between the two solvents is then set to a large value, e.g., *A*_wat,oil_ ≈ 100 in order to reproduce the experimental interfacial tension.^49−51^ Using a larger value for *A*_*ij*_ has the effect of sharpening the transition in the liquid density at the interface. We can reproduce this effect in our model by increasing the repulsion amplitude between water and dodecane beads (both types) from *A*_wat,dod_ = 45 (solid lines in Figure 4a) to 100 (dashed lines in Figure 4a). With this increased repulsion amplitude, we find γ_wat,dod_ = 54.5 ± 0.3 mN m^–1^, which is adjusted to be close to experiment. Such large changes in the oil–water interaction parameters would render our model useless in its ability to reproduce partition coefficients and liquid phase densities for which the model was originally parametrized though. To explore this still a little further, we compare these results from the two DPD models with an MD simulation of a dodecane-water interface (dotted lines in Figure 4a). The resultant MD value of the water–dodecane interfacial tension was γ_wat,dod_ = 56 ± 3 mN m^–1^. We observe (Figure 4a) that in the MD simulation the interface width is even narrower than that in the artificially sharpened DPD model. Thus, we conclude that the underprediction of the oil–water interfacial tension is connected to the overprediction of the width of the interface (Figure 4a).

Given that the interface width in the DPD model is converted to physical units using the length scale mapping, another intriguing possible avenue to improve the match between the model and reality beyond merely increasing *A*_wat,oil_ would be to shrink *r*_c_. This would additionally move the oil–water surface tension in the right direction since γ_wat,dod_ scales as *k*_B_*T*/*r*_c_^2^. The disadvantage of such an approach is that it reduces the level of coarse-graining (e.g., the number of water molecules represented by a water bead), which reduces the physical size of systems that can be studied. A future force-field parametrization exercise that includes interfacial tensions as a target might consider though some combination of reducing *r*_c_ and increasing the alkyl–water bead repulsion amplitudes. We defer further discussion for now but emphasize that the protocols developed here for building chemically specific surface models are not dependent on the particular coarse-grained force field used for the model and are easily transferrable to other models, including those not based on DPD potentials. Further, as the example of the SAM-covered silica surface shows, one can still use the present DPD model provided that the substrate energies are chosen appropriately.

We now outline several avenues for future work. To continue in the above direction, we have started to explore how density functional theory (DFT) may be used to compute the surface energies and density profiles within our wall models, which would obviate the need to undertake any simulations at all. One interesting result of this is that with a simple mean-field DFT, one can devise a wall potential for which the water model (as a fluid of DPD water beads at a specified density) is predicted to be completely structureless away from the wall or, in other words, have a flat density profile. We further find that the required wall potential can be interpreted as corresponding to a “DPD continuum” made from DPD water beads at the bulk water bead density, in the *z* < 0 half space. This provides a theoretical justification for the approach of Goicochea and Alarcón, who first developed this concept.^52^

Additionally, not investigated in the present work, one can ask how the individual molecular groups are distributed in the vicinity of the wall. For example, one might expect that for the pure oil, the terminal −CH_3_ beads in our dodecane model could be preferentially located at the wall, on purely entropic grounds.^53^ Microscopically, the local structure can be controlled to some extent by adjusting the individual DPD bead–wall interactions. In the present work, for simplicity, we did not distinguish between the two hydrocarbon bead types in this respect, but in future works, these could be fine-tuned to reproduce the molecular density profiles in greater detail. Other challenges and limitations posed by realistic-substrate features include representing doped solids, surface defects, and electrostatic interactions that could dominate the nature of the interactions on the real surface.

Finally, our studies could be extended to include the kinetics of wetting, spreading, and detachment. The fluids in our DPD models have well-defined viscosities, and so, in principle, one ought to be able to investigate these dynamical aspects with the current models. In our flat, structureless wall model, though, the wall potential gives rise to forces on the DPD beads which are normal to the surface, and thus, there are no lateral forces. As it stands, therefore, there is nothing to stop the bulk DPD fluid from sliding freely over the wall, which would appear to be completely frictionless. To remove this “feature”, one could introduce lateral wall friction forces along the lines of the dissipative forces between DPD beads, with a corresponding fluctuating wall force to satisfy the fluctuation–dissipation theorem.^54^ With this, one can hope to be able to control and adjust the hydrodynamic slip length at the wall without perturbing the equilibrium structural features such as the surface energies and the contact angle. The movement of the contact line in such models would additionally need to be characterized, and related to the mesoscopic physics of the real system that the model was attempting to emulate.^55^

## Supporting Information Available

Operational definition of the surface energy at a wall such that the Young equation for a droplet on a surface is automatically satisfied and details of results exploring finite size effects for different simulation box sizes (PDF)
