# Supplementary material for: Modeling Contact Angles with Chemically Specific Dissipative Particle Dynamics
Source: Langmuir. 2025 Feb 5;41(6):3877–87. doi: 10.1021/acs.langmuir.4c04023 (PMC11841049; doi:10.1021/acs.langmuir.4c04023)
Supplement: Supplementary file 1 — la4c04023_si_001.pdf [file la4c04023_si_001.pdf]

Supporting Information

Modelling contact angles with chemically specific dissipative particle dynamics

Guadalupe Jiménez-Serratos,[*,†] Patrick B. Warren,[*,†] Scott Singleton,[‡] David J. Bray,[†] and Richard L. Anderson[*,†]

†*The Hartree Centre, STFC Daresbury Laboratory, Warrington, WA4 4AD, UK*

‡*Unilever R&D Colworth Laboratory, Sharnbrook, Bedford, MK44 1LQ, UK*

E-mail: lupe.jimenez-serratos@stfc.ac.uk; patrick.warren@stfc.ac.uk; richard.anderson@stfc.ac.uk

S1 Force balance considerations

In this Appendix to the main article we establish an operational definition of the surface energy at a wall such that the Young equation for a droplet on a surface is automatically satisfied, then confirm the validity of Eq. 7 in the main text, specifying how the wall forces should be incorporated into the appropriate component of the pressure tensor.

Consider a cylindrical droplet sitting on the lower surface of a simulation box, between two confining walls, as shown in Fig. S1. Let the droplet have height H and the contact angle be θ, and consider two planes AA$'$ and BB$'$ extending across the simulation box as indicated in Fig. S1a, with the latter intersecting the droplet at its maximum height. The force across AA$'$ will be $p_1 L_z - 2\gamma_{s1}$ and similarly the force across BB$'$ will be $p_2 H + p_1(L_z - H) - \gamma_{s2} - \gamma_{12} - \gamma_{s1}$, where p_1 and p_2 are the pressures outside and inside the droplet, the corresponding surface energies are γ_{s1} and γ_{s2}, and the interfacial tension is γ_{12}. We suppose that the scale is macroscopic, so that the interfaces are infinitely thin on the length scale of the droplet. Assuming force balance we therefore have $p_1 L_z - 2\gamma_{s1} = p_2 H + p_1(L_z - H) - \gamma_{s2} - \gamma_{12} - \gamma_{s1}$. We note that the pressure inside the droplet exceeds the external pressure by an amount given by the Laplace equation, so that $p_2 - p_1 = \gamma_{12}/R$, where R is the radius of the droplet. The above force balance then simplifies to $\gamma_{s1} = \gamma_{s2} + \gamma_{12}(1 - H/R)$. It follows from elementary Euclidean geometry that if the droplet profile is considered as an arc of a circle, the half-angle subtended by the 'footprint' of the droplet on the surface at the centre of this circle is equal to the contact angle as indicated in Fig. S1b.[1] This implies $H = R(1 - \cos\theta)$, and so we recover the Young equation as given in Eq. 1 in the main text.

The implication is that the total force transmitted across a plane like AA$'$ can be used to make an operational definition of the surface energy γ_z such that the Young equation is satisfied, at least macroscopically. The plane should extend far enough above and below the simulation box to encompass all the forces present. For our purposes, since there are no forces outside the hard reflecting boundaries we need only consider the force transmitted

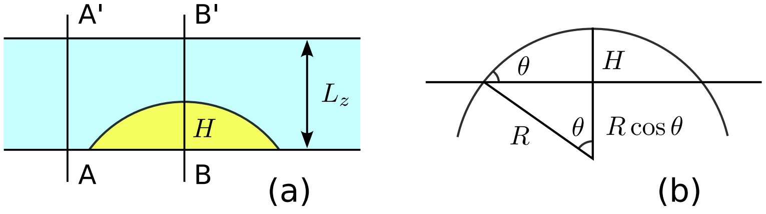

Figure S1: A cylindrical droplet on a surface: (a) the force across AA′ must match the force across BB′; (b) the geometry of the droplet.

across the segment contained between these boundaries.

Now remove the droplet and consider a simulation box containing pure fluid bounded by walls top and bottom, as in the left hand part of Fig. S1a. In this situation we could place the AA′ plane anywhere in the simulation box and we should end up with the same transmitted force, so that we can conclude the force transmitted across such a plane can be written as $L_z\langle P_{xx}\rangle$, where $\langle P_{xx}\rangle$ is the volume-average component of the pressure tensor in the direction parallel to the surface. This now furnishes us with a precise definition of the surface energy, namely $2\gamma_s = L_z(p - \langle P_{xx}\rangle)$, noting there are two walls at the top and bottom of the control volume (Fig. S1a). Since the fluid is isotropic and $\langle P_{xx}\rangle = \langle P_{yy}\rangle$, one can also write this as $2\gamma_s = L_z(p - (\langle P_{xx}\rangle + \langle P_{yy}\rangle)/2)$.

This definition assumes that we know the bulk pressure but in practice we want to avoid having to measure this separately or rely on the barostat. A careful analysis of the force balance normal to the substrate allows us to do exactly this. The starting point is the macroscopic hydrostatic equation, $\partial P_{zz}/\partial z - f_z^{\text{ext}} = 0$, where f_z^{ext} is the external force density exerted by the walls on the fluid. In microscopic terms, this force density is $f_z^{\text{ext}} = \sum_i F_{i,z}^{\text{ext}}\,\delta(\vec{r} - \vec{r}_i) = (1/A)\sum_i F_{i,z}^{\text{ext}}\,\delta(z - z_i)$, where $F_{i,z}^{\text{ext}}$ is the external force exerted by the wall on the i-th DPD bead, and we exploit the fact that on average f_z^{ext} should be independent of position in the lateral direction to replace it by an area average, with $A = L_xL_y$.

Let us integrate the hydrostatic pressure equation, from some point $z = L$ in the bulk

where $P_{zz} \to p$, towards the surface at $z = 0$. We obtain in this way $p = P_{zz}(z) + \int_z^L \mathrm{d}z\, f_z^{\mathrm{ext}}$. Injecting the microscopic expression for the force density into this obtains

$$p = P_{zz}(z) + \frac{1}{A}\sum_i F_{i,z}^{\mathrm{ext}}\,\Theta(z_i - z)\,\Theta(L - z_i)\,, \tag{S1}$$

where the Θ-functions ensure that only DPD beads with $z < z_i < L$ are counted. We integrate once more in the z-direction, using the fact that $\int_0^L \mathrm{d}z\,\Theta(z_i - z) = z_i$ if $z_i < L$, to obtain

$$p = \frac{1}{L}\int_0^L \mathrm{d}z\, P_{zz} + \frac{1}{AL}\sum_i F_{i,z}^{\mathrm{ext}}\, z_i\,\Theta(L - z_i)\,. \tag{S2}$$

As mentioned, the height L in here should be large enough to warrant the assumption that one is in the bulk (where, in fact, $F_{i,z}^{\mathrm{ext}} = 0$ anyway), but not so large as to reach the upper confining wall. However, we can make the exact same argument for this upper confining wall, and combining the expressions yields

$$p = \langle P_{zz}\rangle + \frac{1}{V}\sum_{i,\mathrm{lower}} F_{i,z}^{\mathrm{ext}}\, z_i + \frac{1}{V}\sum_{i,\mathrm{upper}} F_{i,z}^{\mathrm{ext}}\,(L_z - z_i)\,, \tag{S3}$$

noting that the limited range of the wall force obviates the need to retain Θ-functions. Here $\langle P_{zz}\rangle$ is the volume-average component of the conventionally-defined pressure tensor in the z-direction normal to the surface. Treated as an expression for p, this can now be injected into the above definition of the surface energy to obtain the final result

$$\gamma_s = \frac{L_z}{2}\Big(\langle P'_{zz}\rangle - \frac{1}{2}(\langle P_{xx}\rangle + \langle P_{yy}\rangle)\Big)\,, \tag{S4}$$

where, to be explicit,

$$\begin{aligned}
\langle P_{xx}\rangle &= \Big\langle \frac{1}{V}\sum_{i>j} F_{ij,x}(x_j - x_i)\Big\rangle\,, \qquad \langle P_{yy}\rangle = \Big\langle \frac{1}{V}\sum_{i>j} F_{ij,y}(y_j - y_i)\Big\rangle\,,\\
\langle P'_{zz}\rangle &= \Big\langle \frac{1}{V}\sum_{i>j} F_{ij,z}(z_j - z_i)\Big\rangle + \Big\langle \frac{1}{V}\sum_{i,\mathrm{lower}} F_{i,z}^{\mathrm{ext}}\, z_i\Big\rangle + \Big\langle \frac{1}{V}\sum_{i,\mathrm{upper}} F_{i,z}^{\mathrm{ext}}\,(L_z - z_i)\Big\rangle\,.
\end{aligned} \tag{S5}$$

In these $\vec{F}_{ij}$ is the force between the i-th and j-th beads so that the first terms are the conventional virial pressure tensor components. Additionally, $\langle\cdots\rangle$ can be taken to be an ensemble average as well as the indicated volume average. Apart from the formal inclusion of the external forces into P'_{zz}, this is identical to Eq. 7 in the main text and justifies its use for the surface energy at the wall.

The theory leading to these final results in Eqs. (S4) and (S5) rests on the assumptions that there are no DPD beads outside the hard walls, and that the external forces exerted by the walls are only in the z-direction normal to the walls. Without these, such as would be the case where the walls are represented by frozen or tethered beads, the problem would have to be revisited.

Note that the kinetic contributions can be omitted from the pressure tensor components in Eq. (S5). This is justified because the kinetic part of the pressure tensor is isotropic even in an inhomogeneous fluid (the momenta of the DPD beads remain decoupled in the Hamiltonian even if the beads are acted on by an external force). This is also the case for the usual expression for the surface tension of a liquid-liquid interface.

S2 Finite size effects in interfacial tension simulations

Water and dodecane surface energies for various wall interaction strengths, and the water-dodecane interfacial tension, as a function of the initial box size $L_x \times L_y \times L_z$ in an NPT ensemble simulation. All results are in $\mathrm{mN\,m^{-1}}$. The highlighted values are the ones used in the main text. The figure in brackets is an estimate of the error in the final digit.

Table S1: Water surface energies.

$A_{s,\mathrm{wat}}$	$10^2\times20$	$10^2\times50$	$10^2\times200$	$10^2\times400$	$25^2\times20$	$25^2\times40$	$50^2\times20$
10	28.3(2)	28.1(2)	**28.5(4)**	28.4(6)	28.03(5)	28.02(7)	28.05(3)
20	90.1(2)	89.6(2)	**89.8(4)**	88.8(6)	89.92(5)	89.58(7)	89.91(3)
30	125.2(2)	123.9(2)	**123.1(4)**	123.7(6)	125.27(5)	124.09(7)	125.25(3)
40	146.1(2)	144.4(2)	**143.3(4)**	143.3(6)	146.13(5)	144.51(7)	146.09(3)
50	159.9(2)	157.2(2)	**156.4(4)**	157.5(6)	159.90(5)	157.83(7)	159.88(3)

Table S2: Dodecane surface energies.

$A_{s,\mathrm{dod}}$	$10^2\times20$	$10^2\times50$	$10^2\times200$	$10^2\times400$	$25^2\times20$	$25^2\times40$	$50^2\times20$
10	38.3(2)	38.6(3)	**38.5(5)**	40.2(8)	38.15(6)	38.4(2)	38.19(3)
20	103.4(2)	103.0(2)	**102.8(5)**	103.(1)	103.47(7)	103.0(5)	103.39(4)
30	134.7(2)	132.7(4)	**132.8(5)**	132.3(8)	134.8(1)	133.0(1)	134.48(3)
40	151.7(2)	149.9(3)	**147.9(5)**	146.9(8)	151.88(7)	150.0(1)	151.80(4)
50	163.1(2)	160.5(3)	**158.9(5)**	157.5(7)	162.99(6)	160.51(9)	162.92(4)

Table S3: Water-dodecane interfacial tension.

box size	$\gamma_{\mathrm{wat,dod}}$
$10^2\times20$	27.6(2)
$10^2\times50$	27.6(2)
$10^2\times200$	**27.3(4)**
$10^2\times400$	27.2(7)

References

(1) Heath, T. L. *Euclid's Elements*; Dover: New York, 1956; The relevant geometric propositions are from Book III: if a line be tangent to a circle, and from the point of contact a chord be drawn cutting the circle (here, the 'footprint' of the droplet), the angle made by this chord with the tangent (*i. e.* the contact angle) is equal to the angle subtended by the chord in the alternate segment of the circle (Prop. 32), which is equal to the half-angle subtended at the centre of the circle (Prop. 20).
